# Large–scale genetic analysis and biological traits of two SigB factors in *Listeria monocytogenes*: lineage correlations and differential functions

**DOI:** 10.3389/fmicb.2023.1268709

**Published:** 2023-11-13

**Authors:** Pan Mao, Yan Wang, Lin Gan, Lingyun Liu, Jinni Chen, Lingling Li, Hui Sun, Xia Luo, Changyun Ye

**Affiliations:** ^1^National Key Laboratory of Intelligent Tracking and Forecasting for Infectious Diseases, National Institute for Communicable Disease Control and Prevention, Chinese Center for Disease Control and Prevention, Beijing, China; ^2^Department of Bacteriology, Capital Institute of Pediatrics, Beijing, China

**Keywords:** *Listeria monocytogenes*, SigB factor, genetic analysis, lineage, biological trait

## Abstract

**Introduction:**

*Listeria monocytogenes* is a globally distributed bacterium that exhibits genetic diversity and trait heterogeneity. The alternative sigma factor SigB serves as a crucial transcriptional regulator essential for responding to environmental stress conditions and facilitating host infection.

**Method:**

We employed a comprehensive genetic analysis of sigB in a dataset comprising 46,921 *L. monocytogenes* genomes. The functional attributes of SigB were evaluated by phenotypic experiments.

**Results:**

Our study revealed the presence of two predominant SigB factors (SigB_T1_ and SigB_T2_) in *L. monocytogenes*, with a robust correlation between SigB_T1_ and lineages I and III, as well as SigB_T2_ and lineage II. Furthermore, SigB_T1_ exhibits superior performance in promoting cellular invasion, cytotoxicity and enhancing biofilm formation and cold tolerance abilities under minimally defined media conditions compared to SigB_T2_.

**Discussion:**

The functional characteristics of SigB_T1_ suggest a potential association with the epidemiology of lineages I and III strains in both human hosts and the natural environment. Our findings highlight the important role of distinct SigB factors in influencing the biological traits of *L. monocytogenes* of different lineages, thus highlighting its distinct pathogenic and adaptive attributes.

## 1. Introduction

*Listeria monocytogenes* is a facultative pathogenic agent of listeriosis, which is a foodborne infection in the older adults, children, and immunocompromised patients. *L. monocytogenes* demonstrates global distribution with cases throughout Europe, North America, Oceania, Asia, South America, and Africa, highlighting its pervasive presence worldwide (Moura et al., 2021; Koopmans et al., 2023). The annual incidence of listeriosis ranges from 0.1 to 11.3 cases per million individuals, with variations caused by geographical areas and the monitoring techniques that are used (Koopmans et al., 2023). *L. monocytogenes* enters the host and survives a series of potentially lethal conditions in the digestive tract, including gastric acid, biliary salt, and oxidative stress, eventually causing bacteremia, meningitis, and severe maternal–fetal infections (Allerberger and Wagner, 2010). The South African listeriosis outbreak in 2017–2018, caused by ST6 *L. monocytogenes*, resulted in 204 fatalities and over $260 million in fatality-related costs (Olanya et al., 2019). *L. monocytogenes* has been found in a diverse range of sources, with a notable predominance in contaminated food products such as processed meats, soft cheeses, raw milk, and ready-to-eat foods (Ryser and Marth, 2007). This pathogen is also extensively found in natural environmental reservoirs, such as soil and polluted water systems (Ryser and Marth, 2007).

*Listeria monocytogenes* can be classified into 4 lineages, 13 recognized serotypes, and numerous sequence types (STs). Certain clones of *L. monocytogenes* have been verified and are more closely linked to clinical cases or food sources. Lineage I strains are used to form a majority of clinical isolates, while lineage II strains exhibits a more general distribution in food isolates (Maury et al., 2016). ST1, ST2, ST4, and ST6 clones are linked to clinical isolates, while ST9 and ST121 clones are common isolates in food sources (Maury et al., 2016; Muchaamba et al., 2021). Additionally, ST87 isolates have been identified as a clinical clone type in China (Wang et al., 2015; Li et al., 2018). The molecular characteristics of *L. monocytogenes* have been shown to be highly divergent in food surveillance, clinical cases, animals, and environment (Maury et al., 2016; Muchaamba et al., 2021). Although some genetic elements in a specific group were associated with virulence and/or stress resistance phenotypes, such as LIPI-4 in CC4, LGI-1 in CC8, SSI-2 in ST121, and pLMST6 in CC6, the underlying causes behind the observed epidemiological distribution and phenotypic variations in *L. monocytogenes* remain unexplained (Bergholz et al., 2018).

As a facultative bacterial pathogen, *L. monocytogenes* possesses remarkable regulatory networks in response to various conditions, such as abrupt environmental changes and host-induced stresses (Guariglia-Oropeza et al., 2014). The transcriptional regulation in bacteria serves as a crucial mechanism for their rapid adaptation and reproduction in an unpredictable environment. Sigma factors are initiation factors that induce the binding of RNA polymerase to promoters, thereby enabling cells to precisely regulate the timing and specificity of gene expression (Feklistov et al., 2014). As a central regulator, the sigma factor B (SigB) has been demonstrated to regulate a factor consisting of approximately 300 genes related to stress response, pathogenesis, cellular homeostasis, and metabolism in *L. monocytogenes* (Chaturongakul et al., 2011; Liu et al., 2017).

Analysis of *sigB* in 6 *Listeria* species comprising 4,390 isolates showed that non-synonymous mutations occurred less frequently than synonymous mutations in 164 *sigB* allelic types (Liao et al., 2017). The regulator *sigB* is a relatively stable gene in *Listeria* that has not significantly undergone evolution due to positive selection and homologous recombination (Liao et al., 2017). However, as the regulatory center of large functional networks, SigB has several characteristics that have not been explored yet, such as the specific types of *sigB* alleles and their unknown biological significance.

In this study, we conducted a large-scale genetic analysis of the *sigB* gene in 46,921 *L. monocytogenes* isolates and associated *sigB* with background information about its host bacteria. To evaluate the biological function of two dominant SigB factors, we further performed chromosomal mutation modification, cell culture assays, and survival phenotype analysis.

## 2. Materials and methods

### 2.1. Genome sequence of *L. monocytogenes* isolates

This study included whole genome data of 46,921 *L. monocytogenes* isolates from the NCBI database (Supplementary Table S1). The geographic information and isolation source of *L. monocytogenes* were obtained from the NCBI BioSample database. To determine the sequence types (STs) and lineage of *L. monocytogenes, in silico* MLST analysis was performed using allele scheme information from the BIGSdb database (Moura et al., 2016).

### 2.2. Bioinformatics analysis of SigB protein

For homology analysis, BLASTN was performed to match for *sigB* with each *L. monocytogenes* genome in translated CDS format, followed by *in silico* extraction and translation. Corresponding to the reference strain EGD-e, the complete *sigB* gene was classified as having 259 amino acids. The distinct amino acid residues within the full-length SigB were identified through the alignment of protein sequences. Each dissimilar amino acid residue signifies a unique type of SigB factor. Those with fewer amino acids were categorized as premature stop codon (PMSC) variants. The protein family and conserved protein domains were performed with the UniProt and Pfam databases (Mistry et al., 2021; UniProt, 2021). The graphical representation of the amino acid probabilities at each site was generated by WebLogo (Crooks et al., 2004).

### 2.3. Bacterial strains and growth conditions

The experimental strains *L. monocytogenes* ICDC-LM188 (LM188) (Lineage I) and EGD-e (Lineage II) were used in this study. Strains were normally cultured in brain heart infusion (BHI, Oxoid, UK) broth or agar at 37^o^C. Modified Welshimer's broth (MWB, HiMedia, India) was used as a minimally defined medium (Premaratne et al., 1991). Chloramphenicol (Solarbio, China) was added to the culture medium when required at a concentration of 10 μg/ml.

### 2.4. Chromosomal modifications of *sigB* in *L. monocytogenes*

Reference strain EGD-e and experimental strain LM188 are used in this study. EGD-e is a reference strain (ATCC BAA-679) of lineage II and is widely used in scientific reports, and the LM188 strain belongs to the prevalent genotype of lineage I in China (Wang et al., 2019). The *sigB*_*T*1_ gene of wild-type *L. monocytogenes* LM188 (Linage I) was replaced by *sigB*_*T*2_ through homologous recombination, while the *sigB*_*T*2_ gene of the wild-type EDG-e (Linage II) strain was replaced by *sigB*_*T*1_. The substituted codons, adjunct fragments from WT codons, and restriction sites (*Sal*I and *BamH*I) were combined *via* DNA synthesis in Tsingke Biotechnology Co., Ltd. Each 1,616-bp linear product was cloned into vector pKSV7, resulting in the pKSV7::*sigB*_T1_ and pKSV7::*sigB*_T2_ vectors, which were then transformed into electrocompetent EDG-e and LM188 cells (Zhang et al., 2018). The electroporated cells were spread on BHI plates with chloramphenicol at 37°C, followed by passage cultivation at 42°C for homologous recombination. The plasmid pKSV7 was cured after growth at 37°C in BHI broth. The chromosomal *sigB* mutation strains LM188_MT_T2_ and EGD-e_MT_T1_ were produced through homologous recombination and verified by sequencing.

### 2.5. RNA extraction and relative expression quantity of *sigB*

The total RNA was extracted with TRIzol (Invitrogen) and then transcribed into cDNA using Fastking GDNA Dispelling RT Supermix (Tiangen). The expression of *sigB* was measured by real-time PCR using SuperReal PreMix Plus (Tiangen). The expression level of *sigB* relative to that of the housekeeping gene (16S *rDNA*) was assessed by the comparative 2^−ΔΔ*CT*^ method.

### 2.6. Cytotoxicity and invasion assays

The cytotoxic effect was evaluated on the human colon adenocarcinoma cell line HT-29, according to the previous method (Gan et al., 2020). Cytotoxicity assays were performed by measuring the release of lactate dehydrogenase (LDH) as an indicator of cell injury. HT-29 (4^*^10^4^) cells were seeded in 96-well plates with an infection of bacteria at an MOI of 50 bacteria. After 4 h of incubation, the LDH concentration in the cell supernatants was determined according to the manufacturer's instructions of CytoTox 96^®^Non-Radioactive Cytotoxicity Assay (Promega, USA).

For invasion assay, HT-29 cells (2^*^10^5^) were infected with bacteria at an MOI of 50 on 24-well plates (Gan et al., 2020). After 1 h of infection, the cells were treated with 50 μg/ml of gentamicin (Solarbio, China) for 30 min and washed with DMEM (Gibco, USA). The cells were then lysed with 0.1% Triton X100 (Sigma, USA). The intracellular bacteria were incubated on a BHI plate for colony enumeration.

### 2.7. Biofilm formation and cold tolerance assays

For *in vitro* growth assays, the *Listeria* isolates were initially cultured in BHI broth for the activation of bacteria at a dilution ratio of 1:100. Subsequently, a bacterial suspension with a log-phase concentration of 10^7^ CFU was transferred and incubated in MWB at 30°C. The optical density of the culture at 600 nm (OD600) was recorded at a 1-h interval using a Bioscreen C microbiology reader. The experiment was repeated three times, with five replicate wells for each trial.

Biofilm formation was quantified using the crystal violet staining method (Mao et al., 2021). Approximately 2^*^10^7^ CFU of fresh bacterial suspensions were initially spread on 96-well microplates. The plates were incubated at 30°C for biofilm formation. The liquid and non-adhered cells were removed and then gently rinsed three times with PBS. The biofilm was stained with 1% crystal violet and then released with 95% ethanol. The total biofilm biomass was evaluated by measuring the OD value at 595 nm. Each experiment was replicated three times for three independent experiments.

Approximately 2^*^10^7^ CFU of fresh bacterial suspension was transferred to 1 ml of MWB medium and prepared to be kept for long-term preservation at 4°C for 60 days. Following thorough vortex mixing, the bacteria were cultured on a BHI agar plate for colony enumeration. Three independent experiments were conducted with three replicates.

### 2.8. Statistical analysis

The relationship between the *L. monocytogenes* lineage and the type of SigB factor was analyzed by Fisher's exact test. The OD_600_ was assessed by repetitive measurement deviation analysis. Invasion efficiency, LDH concentration, and biofilm biomass used t-tests for statistical analysis. Significant differences were judged at a *P* < 0.05, *P* < 0.01, or *P* < 0.001.

## 3. Results

### 3.1. Genetic analysis and protein domain prediction of *sigB* gene in *L. monocytogenes*

The *sigB* gene is composed of 780 base pairs (Bp) that code for 259 amino acids of full-length protein. Through the sequence analysis of 46,921 *L. monocytogenes* isolates, 46,773 isolates (99.79%) contain a single full-length *sigB* gene, while 86 isolates contain premature stop codons (PMSCs) in the *sigB* gene. Additionally, the *sigB* gene could not be aligned with the genomic sequences of 12 isolates, and 4 isolates harbored two copies of the *sigB* gene. Furthermore, the *sigB* gene in 46 isolates was fragmented and not included in the analysis (Supplementary Table S1).

**Figure 1:**
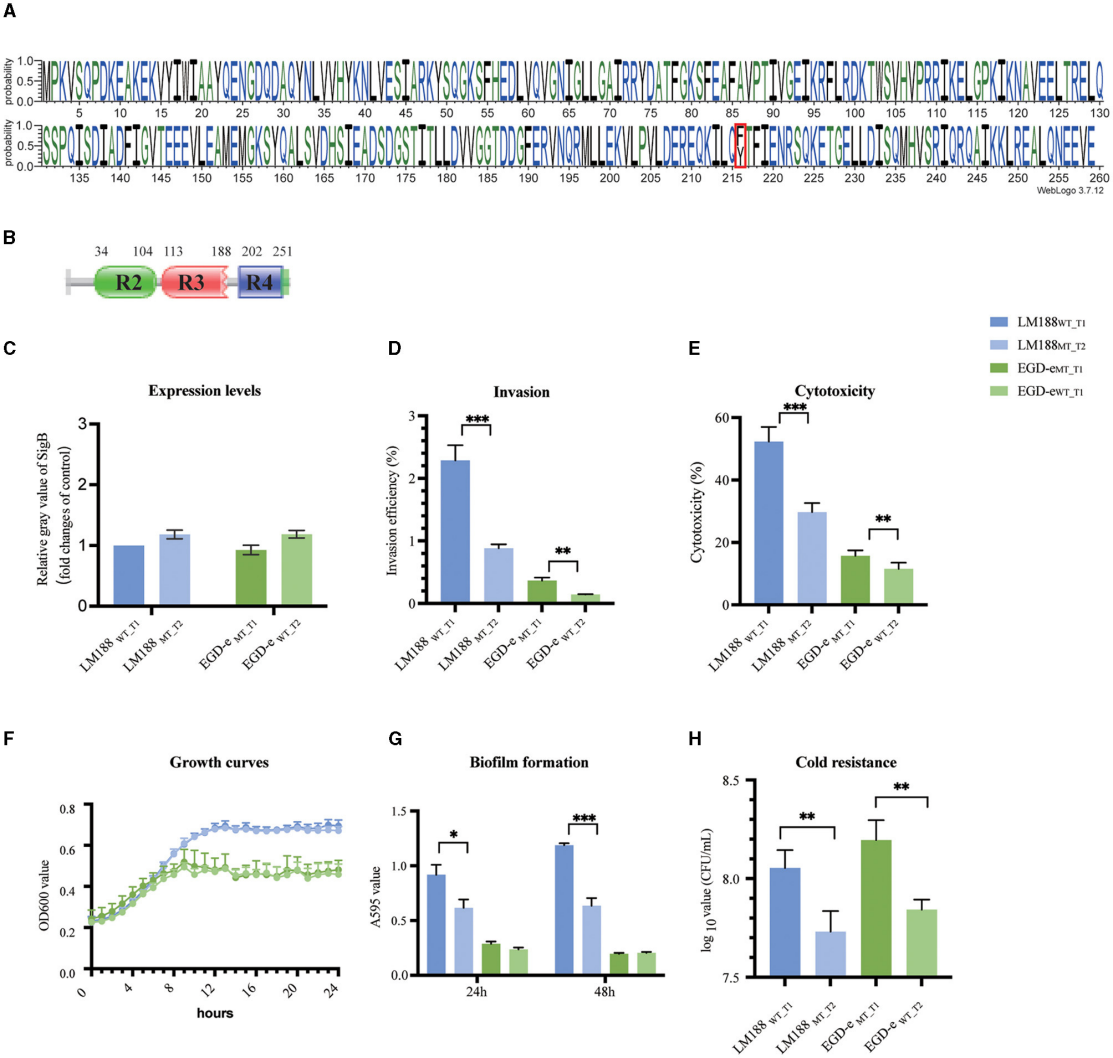
**(A)** Weblogo built using 46,773 sequences of full-length SigB. **(B)** Conserved protein domains. **(C)** Expression levels. **(D)** Invasion. **(E)** Cytotoxicity. **(F)** Growth curves. **(G)** Biofilm formation. **(H)** Cold resistance (^*^P < 0.05, ^**^P < 0.01, ^***^P < 0.001).

Among full-length *sigB* genes, 112 distinct protein types of SigB factor were identified and characterized by 88 variant sites and 171 completely conserved sites, and the predominant types were SigB_T1_ and SigB_T2_ (Figure 1A, Supplementary Tables S1, S2). In addition, the *sigB* gene of 86 *L. monocytogenes* isolates was truncated with deletion mutations in 43 isolates, insertion mutations in 20 isolates, and nonsense point mutations in 23 isolates (Supplementary Table S1). Finally, one strain of an unknown lineage carried both SigB_T1_ and SigB_T2_ factors, and three isolates belonging to lineage II carried two identical SigB_T2_ factors (Supplementary Table S1).

**Figure 2:**
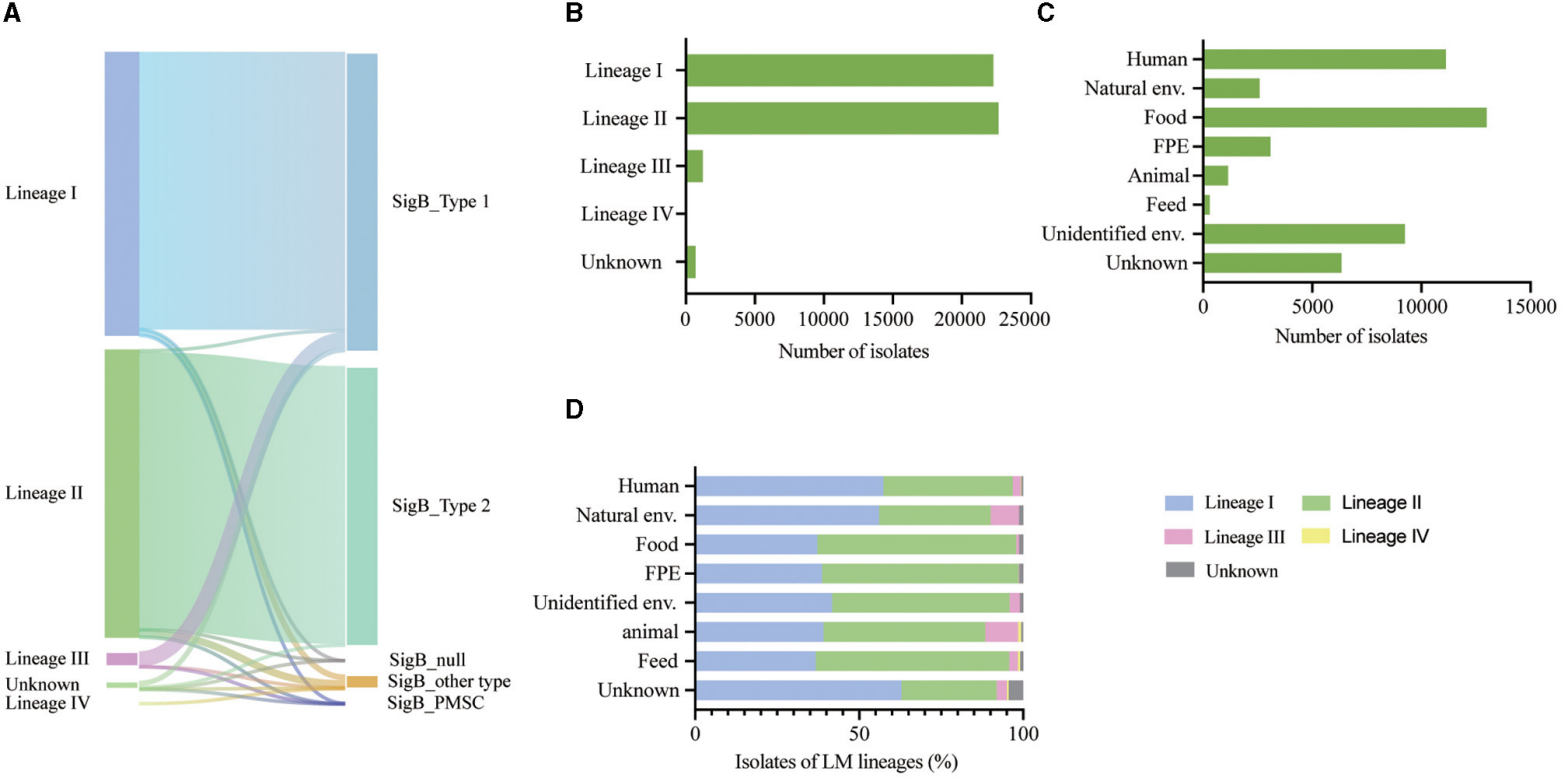
**(A)** Sankey diagram illustrating the correlation between variation type of SigB and lineages. **(B)** Prevalence of *L. monocytogenes* categorized by lineages. **(C)** Prevalence of *L. monocytogenes* based on different sources. **(D)** Proportional distribution of lineages among *L. monocytogenes* (LM) from diverse sources.

The analysis revealed two predominant types of SigB factors, namely, SigB_T1_ (50.42%) and SigB_T2_ (47.09%). Both types shared a variant site at codon 216. Furthermore, SigB_T1_ encodes phenylalanine at this codon, while SigB_T2_ encodes tyrosine (Figure 1A). The SigB protein belongs to the Sigma 70 family and contains three domains, including Sigma 70 r2, r3, and r4, which are predicted by the UniProt and Pfam databases (Figure 2B). The variant site of codon 216 is located on the r4 domain.

### 3.2. SigB_*T*1_ outperforms SigB_*T*2_ in enhancing cellular invasion, cytotoxicity, biofilm formation, and cold tolerance

To assess the difference between the biological functions of SigB_T1_ and SigB_T2_, we constructed *sigB* mutants in chromosomal loci by homologous recombination. Four strains of *L. monocytogenes*, namely, wild-type LM188_WT_T1_, mutant-type LM188_MT_T2_, wild-type EGD-e_WT_T2_, and mutant-type EGD-e_MT_T1_, were used for conducting phenotypic experiments. Additionally, there was no significant difference in *sigB* gene expression levels between LM188_WT_T1_ and LM188_MT_T2_, as well as between EGD-e_WT_T2_ and EGD-e_MT_T1_ under BHI conditions (Figure 1C).

Cellular invasion and cytotoxicity assays in the HT-29 cell line were assessed to evaluate the effect of two SigB types on virulence. LM188_WT_T1_ strain exhibited significantly higher invasion ability (*P* < 0.001) and cytotoxicity (*P* < 0.001) compared to the LM188_MT_T2_ strain. Additionally, the EGD-e_MT_T1_ strain also showed significantly higher invasion capacity (*P* < 0.01) and cytotoxicity (*P* < 0.01) than the EGD-e_WT_T2_ strain (Figures 1D, E). These results suggest that SigB plays a significant role in the virulence of *L. monocytogenes*, and SigB_T1_ outperforms SigB_T2_ in enhancing cellular invasion and cytotoxicity.

Biofilm formation and proliferation ability were evaluated for culture in a minimally defined MWB medium. No significant disparity was observed in the growth profiles between LM188_WT_T1_ and LM188_MT_T2_, as well as between EGD-e_MT_T1_ and EGD-e_WT_T2_ (Figure 1E), suggesting that the variation in SigB factor has no significant effect on bacterial growth. We found that the LM188_WT_T1_ strain exhibited higher biofilm formation ability at 24 h (*P* < 0.05) and 48 h (*P* < 0.001) than the LM188_MT_T2_ strain, and both EGD-e_MT_T1_ and EGD-e_WT_T2_ strains had weak growth and biofilm formation ability in MWB medium (Figures 1F, G). Furthermore, the results also showed that LM188_WT_T1_ and EGD-e_MT_T1_ strains with SigB_T1_ exhibited stronger cold tolerance compared to LM188_MT_T2_ and EGD-e_WT_T2_ carrying SigB_T2_ at 4°C (*P* < 0.01) (Figure 1H). These results suggest that the SigB type has a significant effect on biofilm formation and cold tolerance of *L. monocytogenes*, and SigB_T1_ exhibits superior performance compared to SigB_T2_ in terms of biofilm formation and cold tolerance.

### 3.3. Correlation of SigB_*T*1_ and SigB_*T*2_ with lineages and their distribution in *L. monocytogenes*

We analyzed 46,921 *L. monocytogenes* genomes from 6 continents, 66 countries, and various sources, including 22,288 lineage I isolates, 22,644 lineage II isolates, 1,227 lineage III isolates, 65 lineage IV isolates, and 697 unknown lineage isolates (Figures 2B, C).

After analyzing 46,921 *L. monocytogenes* isolates from the NCBI database, it was observed that SigB_T1_ was present in 21,791 isolates (97.77%) of lineage I, 1,188 isolates (96.82%) of lineage III, 207 (0.91%) isolates of lineage II, and 400 (57.38%) isolates of an unknown lineage. SigB_T2_ existed in 21,786 (96.21%) isolates of lineage II and 235 (33.72%) isolates of unknown lineage. Therefore, SigB_T1_ is highly correlated with lineages I and III (*P* < 0.001), and SigB_T2_ is highly correlated with lineage II (*P* < 0.001) (Figure 2A).

The *L. monocytogenes* isolates were distributed globally and there was an association between *L. monocytogenes* lineages and different sources. Isolates from human and natural environmental sources were distributed across the lineages, with lineage I being the most prevalent (Figure 2D). Similar proportions of each lineage were distributed in food and food production environment (FPE)-sourced isolates, with lineage II being the most prevalent (Figure 2D).

## 4. Discussion

*L. monocytogenes* is an important foodborne pathogen that can colonize and penetrate the gastrointestinal tract, blood–brain barrier, and placental barrier. *L. monocytogenes* develops numerous stress response mechanisms in order to survive and persist in a variety of harsh environments, both outside and within the host. SigB, an alternative general stress sigma factor, modulates the transcriptional landscapes of stress response, pathogenesis, and homeostasis in *L. monocytogenes* (Garner et al., 2006; Toledo-Arana et al., 2009; van Der Veen and Abee, 2010; Gahan and Hill, 2014; Dorey et al., 2019). Transcription factors bind to the specific DNA sequences of the target gene and then control the initiation or inhibition of their transcription, regulating the gene expression in bacteria (Balleza et al., 2009). Mutations of bacterial transcription factors, such as K259 lysine acetylation of HrdB, F318A mutation of σ54, I48S in region 1.1 of *E. coli* σ70, and dynamic variation of MarR transcription factors, affect transcription regulation by altering their interaction with the RNAP core enzyme and its binding activity toward target promoters (Wigneshweraraj et al., 2002; Deochand and Grove, 2017; Kim et al., 2020; Pletnev et al., 2020). The novel SigB (Q225P) mutation in *Staphylococcus aureus* maintains virulence but enhances biofilm formation by affecting the promoter activity (Liu et al., 2018). The study of variation in transcription factors in bacteria can offer important insights into gene expression regulation and bacterial responses.

In this study, we demonstrated that the correlation between SigB_T1_ and lineage I/III as well as SigB_T2_ and lineage II, and codon 216 of SigB play an important role in the invasion and environmental stress adaptation of *L. monocytogenes*. The 216 variant site of SigB is located in domain sigma70 r4, which forms the second largest interface with RNA polymerase and serves as a key contact point for transcriptional activators that recognize the upstream sequences of the -35 promoter region (Paget, 2015).

In this study, 92.39% of isolates were classified as lineage I in *L. monocytogenes* isolates carrying SigB_T1_, and 98.93% of the isolates were identified as lineage II among the isolates harboring SigB_T2_. We found that *L. monocytogenes* with SigB_T1_ had a higher invasive ability to human colon cancer cells than isolates with SigB_T2_, suggesting that SigB_T1_ is associated with the high virulence of *L. monocytogenes*. When *L. monocytogenes* reaches the host intestinal lumen, transcriptional reshaping is activated by SigB-mediated virulence genes (Toledo-Arana et al., 2009). SigB-mediated control of *inlA* and *inlB* expression is critical for *L. monocytogenes* invasion of host epithelial cells, as demonstrated by a significantly reduced invasion in a *sigB* deletion mutant strain (Kazmierczak et al., 2003; Kim et al., 2005; Garner et al., 2006; Bierne et al., 2007). InlA is essential for the pathogenesis of *L. monocytogenes* as it mediates the identification and invasion of epithelial cells through its specific interaction with E-cadherin, which is a fundamental step to crossing the intestinal barrier (Bierne et al., 2007; Disson et al., 2008). Among 15 transcriptional regulators, SigB is a key factor in the invasion of human epithelial colorectal adenocarcinoma cells by *L. monocytogenes* (Rukit et al., 2022). Therefore, SigB_T1_ is associated with the prevalence of lineage I and lineage III of *L. monocytogenes* in patients and animals.

Researchers in the field of the molecular epidemiology of *L. monocytogenes* have focused on investigating the source of human infection as well as its presence in food and food processing environments, with lineage I being frequently associated with human infection and lineage II being prevalent in food and environmental sources (Maury et al., 2016; Koopmans et al., 2023). However, little attention has been paid to its prevalence in natural environments. This study revealed that *L. monocytogenes* isolates in natural vs. food environments have distinct molecular characteristics, with lineage I being more prevalent in natural environments and lineage II being more prevalent in food environments. Additionally, in this study, a substantial proportion of lineage III strains originated from natural environments and animal sources. These are likely due to two distinct environmental conditions: natural environments have limited nutrition, while food environments have abundant nutrition.

The *sigB* gene plays a crucial role in biofilm formation and has increased expression levels in both static and continuous-flow biofilms (van Der Veen and Abee, 2010; Hsu et al., 2020). Our findings suggest that SigB_T1_ enhances biofilm formation in low-nutrient settings in the MWB culture medium. The effect of SigB factor types on clinical strain LM188 is significantly apparent compared to that on strain EGD-e. This might be attributed to weaker phenotypes of EGD-e, which is a strain of unclear source, specifically biofilm formation and reduced growth in defined minimal media. Therefore, SigB_T1_ contributes to the higher biofilm-forming ability observed in lineage I strains under such conditions, which may partially explain its prevalence and persistence in natural environments.

The *sigB* gene is involved in survival from chill stress in *L. monocytogenes*, with varying degrees of impact influenced by different serotypes (Moorhead and Dykes, 2004). Our finding also indicates that SigB_T1_ isolates exhibit a greater capacity for cold tolerance as compared to SigB_T2_ isolates when cultured in a minimal medium. This observation suggests that SigB_T1_ would help lineage I and lineage III isolates endure low temperatures under nutrient-poor conditions and increase their likelihood of survival and persistence in frigid natural environments. *L. monocytogenes* ubiquitously exist in various sources and can survive in the presence of high osmolarity, low-temperature conditions, and extreme environments (Chan and Wiedmann, 2009). Different microbial lineages have adapted to different environments and sources, and their proportions vary depending on the environment they inhabit.

In conclusion, our study demonstrated that lineage-specific SigB types influenced the survival, virulence, and adaptation of *L. monocytogenes* under specific conditions. Contrary to SigB_T2_, SigB_T1_ exhibited a more pronounced role in promoting invasiveness and virulence as well as improving biofilm formation and long-term cold survival under limited nutritional conditions. SigB_T1_ could contribute to the prevalence and persistence of *L. monocytogenes* linage I and linage III strains in clinical settings and the natural environment, highlighting its pivotal role in the epidemiology of this pathogen. These findings offer a new insight into the molecular mechanisms underlying the phenotypic variations and epidemiological distribution of *L. monocytogenes*, paving the way for future research on the development of targeted interventions to prevent and control infections caused by this pathogen.

## Data availability statement

The datasets presented in this study can be found in online repositories. The names of the repository/repositories and accession number(s) can be found in the article/Supplementary material.

## Author contributions

CY: Conceptualization, Funding acquisition, Project administration, Resources, Supervision, Validation, Writing—review & editing. PM: Conceptualization, Formal analysis, Methodology, Software, Visualization, Writing—original draft. YW: Supervision, Project administration, Writing—review & editing. LG: Data curation, Writing—review & editing. LLiu: Methodology, Writing—original draft. JC: Methodology, Writing—original draft. LLi: Methodology, Writing—original draft. HS: Project administration, Writing—review & editing. XL: Project administration, Writing—review & editing.
